# High-resolution dataset for tea garden disease management: Precision agriculture insights

**DOI:** 10.1016/j.dib.2025.111379

**Published:** 2025-02-12

**Authors:** Rimon, Sajib Bormon, MD Hasan Ahmad, Sohanur Rahman Sohag, Amatul Bushra Akhi

**Affiliations:** Department of Computer Science and Engineering, Daffodil International University, Dhaka, Bangladesh

**Keywords:** Tea leaf dataset, Image identification, Image preprocessing, Agriculture, Transformer, Deep learning, and Computer vision

## Abstract

The economic development of many countries largely depends on tea plantations that suffer from diseases adversely affecting their productivity and quality. This study presents a high-resolution dataset aimed at advancing precision agriculture for managing tea garden diseases. The size of the dataset is 3960 images and pixel dimension is (1024 × 1024) of the images were collected by using smartphones. This dataset contains detailed images of Tea Leaf Blight, Tea Red Leaf Spot and Tea Red Scab maladies inflicted on tea leaves as well as environmental statistics and plant health. The images were captured and stored in JPG format. The main aim of this dataset is to provide tool for detection and classification of different types of tea garden disease. Applying this dataset will enable the development of early detection systems, best-practice care regimens, and enhanced general garden upkeep. A range of images presenting the most prevalent diseases afflicting tea plants are paired with images of healthy leaves to provide a comprehensive overview of all the circumstances that can arise in a tea plantation. Therefore, it can be used to automate diseases tracking, targeted pesticide spraying, and even the making of smart farm tools with development of smart agricultural tools hence enhancing sustainability and efficiency in tea production. This dataset not only provides a strong foundation for applying precision techniques in tea cultivation in agriculture, but also can become an invaluable asset to scientists studying the issues of tea production.

Specifications Table

**Table:** 

Subject	Computer Science
Specific subject area	Disease classification, Image Preprocessing,Images categorization,Image Detections*.*
Type of data	Image.
Data collection	Between October and December 2023, some high-resolution images of tea plants were captured from two different garden in Moulvi Bazar, Sylhet, Bangladesh. The project was conducted under the supervision of an expert from Bangladesh's Ministry of Agriculture.
Data source location	**Location:** Moulvi Bazar tea garden,Sylhet **Country:** Bangladesh
Data accessibility	Repository name: Mendeley Data Data identification number: 10.17632/tt2smzrzrs.4 Direct URL to data: https://data.mendeley.com/datasets/tt2smzrzrs/4
Related research article	None

## 1 Value of the Data

• Tea is a major global agricultural crop with economic implications as well as cultural significance. This drink is famous for diverse tastes and health benefits. In many civilizations, tea remains their main beverage [1]. There are several countries that supply most of the world's tea including India, China, Sri Lanka and Bangladesh [2]. Nevertheless, numerous diseases can affect it leading to reduced quality and yield hence threatening its output [3].
• Tea gardens are one setting where advances in image processing and machine learning have produced new prospects for early detection and intervention in various diseases [4]. Tea red scab, Tea red leaf spot, Tea leaf blight and Healthy classes are included in the study; therefore, giving a holistic understanding of tea plant health.
• This dataset is important because it captures a wide range of environmental variables, providing critical insights into seasonal and locational variations that might impact the study's results. We have gathered high-resolution data at two different locations in Moulvi Bazar during two different seasonal periods (Spring and Summer). By capturing variations and ensuring a comprehensive dataset, this dual-location, multi-seasonal approach aims to improve the robustness and applicability of the dataset. By offering a variety of environmental contexts, this dataset enhances the study and is crucial for extrapolating the results.
• Higher severity diseases, such as Tea Red Scab, Tea Red Leaf Spot, and Tea Leaf Blight, which are more harmful than other tea leaf diseases, are the focus of this dataset [5]. Early detection of these diseases is essential for efficient disease management and general plant health as they have a substantial impact on tea yield and quality. The dataset prioritizes severe diseases in order to facilitate early detection, which is crucial for nurturing the health of tea plants and reducing financial losses.
• The dataset is subjected to rigorous preprocessing steps such as image segmentation, noise removal, scaling and normalization. The dataset was designed with quality and consistency in mind. We have resorted to data augmentation techniques to balance class distributions and improve the dataset as a result leading into development of robust deep learning models. This demanding technique ensures that the dataset is reliable and applicable under real life circumstances.
• To this end, this research aims at introducing an approach for handling tea garden diseases through deep learning as well as advanced image processing. Thus, early detection of diseases can lead to increased productivity and sustainability of tea gardens [6]. This paper offers insightful analysis together with tools for field researchers and practitioners thereby emphasizing on transformative potentialities of modern agricultural technologies.
• The larger agricultural community can find this work as a useful tool and realize the important role that computer vision and deep learning can play in modern agricultural practices. The proposed dataset and approaches help to develop precision agriculture through more efficient and effective disease control techniques in tea gardens. This helps not only the tea business but also the worldwide initiatives for environmentally friendly farming methods.
• We have collected our dataset from two garden as well as considering two seasons. It is carefully chosen with high-quality images capturing even the most faint indications of (Leaf Blight, Tea Red Leaf Spot and Tea Red Scab) tea leaf diseases. The well-balanced dataset guarantees enough representation of all diseases types, therefore improving the accuracy and dependability of any constructed predictive model. Furthermore, material comes from several geographical areas, which enables a more complete knowledge of disease trends in many climates and surroundings. This variety makes our dataset extremely generalizable and useful for research and management of tea leaf diseases in the real world.
  Some key points are given highlighting the dataset's impact on advancing tea plantation productivity and quality:
  1. Early Disease Detection: Facilitates accurate and timely identification of diseases and pests, preventing large-scale damage to crops.
  2. Improved Quality Control: Enhances the ability to monitor plant health and implement targeted interventions, ensuring higher-quality tea leaves.
  3. Data-Driven Decisions: Provides actionable insights for farmers, replacing traditional methods with evidence-based strategies.
  4. Scalability: Supports the automation of monitoring and management practices.

## 2 Background

This dataset was gathered in order to address the challenge of identifying the different stages of development of common horticultural tea plant diseases. The dataset's development aligns well with current precision agriculture initiatives, which use technological advancements to enhance agricultural practices. According to this perspective, the absence of large datasets about diseases affecting tea plants delayed the creation of accurate detection algorithms and hence contributed to innovation. We have collected 3960 high-resolution images displaying several states: Healthy (2270 images), Tea Leaf Blight (509 images), Tea Red Leaf Spot (561 images), and Tea Red Scab (620 images). This dataset enables fast and accurate disease identification impacting tea plants and is a valuable tool for validation and training of deep learning algorithms. Through this paper, researchers will have easier access to an appropriate dataset that will improve transparency, repeatability, and the potential for further research to optimize agricultural processes. This work helps to build more accurate and dependable models by providing a well-curated dataset, therefore advancing agricultural research and practices.

## 3 Data Description

The dataset contains images of both diseased and healthy leaves, showing different stages of tea plant development. Using the cameras of a Vivo V21(64 megapixel sensor) and a Redmi Note 11(50 megapixel sensor) smartphone, these images were manually captured between October 2023 and the end of 2023 from the Moulvi Bazar tea garden in Sylhet, Bangladesh, under the supervision of a subject matter expert. The resulting 1024 × 1024 pixel images are taken and stored in JPG format. The images of the collection are labelled with the related disorders, which simplifies analysis and classification.

Challenges Created During collecting data:

1. Noisy locations and Uneven Lighting: Our biggest challenge while collecting data was taking images in noisy locations and with uneven lighting.
2. Overlapping leaves and Dense Vegetation: It was difficult to obtain a clear and distinct image of a specific leaf due to the overlapping leaves and dense vegetation of the tea plants.
3. Variety in Leaf Size and Shape: The variety in leaf size and shape made precise placement and framing essential to preserve dataset consistency.
4. Glare and Reflections: It was harder to take images because of the glare and reflections the glossy leaves of the tea trees produced.
5. Pest Presence: Sometimes the quality and utility of the images deteriorated due leaf pest presence.

**Fig. 1:**
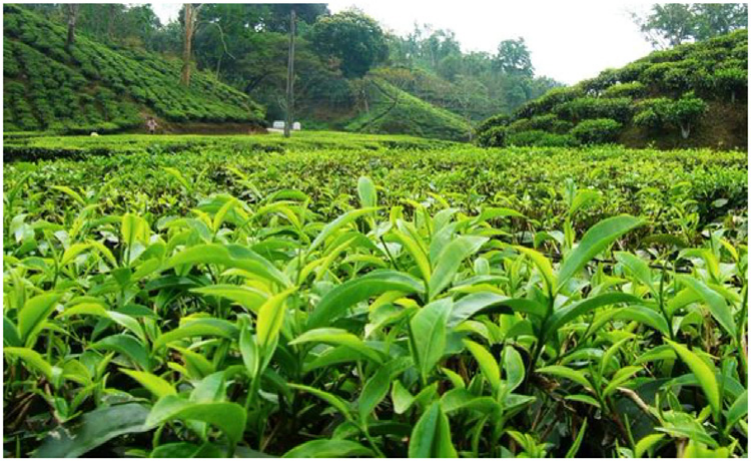
The Moulvi Bazar tea garden, where most of the dataset images were collected.

Fig. 1 shows the field of the Tea Garden from whence we collected dataset images.

Four varieties of tea plant leaves are shown in this study: healthy, tea red scab, tea red leaf spot, and tea leaf blight. Table 1, Table 2 details on these several kinds of tea leaves.

**Table 1:** Details in the dataset on the several types of tea leaf disease.

Modern agricultural research tools like automation offers several advantages for a country's agriculture sector [7]. The principal benefit is the improved quality. Automation task like disease type identification in tea plants guarantees a consistent and very competent final result [8]. This is absolutely necessary to meet the demands of consumers and international marketplaces, which frequently have strict quality standards [9,10]. Though personally detecting the condition is still relatively prevalent, it is well acknowledged to have certain disadvantages [11]. Due to its subjectivity and dependence on variables like personal judgment and weariness, human observation is subject to mistakes and disagreements. Additionally, it requires a lot of time, particularly when handling more leaves, which could lead to inefficiencies and increased labor costs [12]. By means of computer vision algorithms, these systems automatically detect and assess diseases depending on many quality criteria [13]. Computer vision lets one carefully measure and investigate properties including colour, size, and form [14]. Using computer vision helps the process to be more accurate, efficient, and scalable, therefore guaranteeing that the agricultural methods satisfy the high criteria needed by consumers and international markets [15,16].

In this study, a dataset is presented to enable these developments. Our dataset is titled Tea Plant Disease Classification Dataset. This dataset folder is divided into four subdirectories: Healthy, Tea Leaf Blight, Tea Red Leaf Spot and Tea Red Scab. Images of tea plant leaves arranged by condition abound in every folder. The dataset consists of the augmented images produced with data augmentation methods in addition to the original ones. The folder containing the Tea Plant Disease Classification Dataset requires 114 MB.

In this study, we utilised self-capturing images of tea plant leaves captured with a mobile phone. In the first phase, an overall of 3960 images were collected, which corresponded to four distinct classes: 509 images of tea leaf blight, 561 images of tea red leaf spot, 620 images of tea red scab, and 2270 images of healthy leaves. Following the augmentation, each class has 1000 images and we have produced 4000 images overall. These data follow JPG format with 1024 × 1024 pixels in RGB format.

## 4 Experimental Design, Materials and Methods

### 4.1 Camera specification

The Redmi Note 11 and Vivo V21 smartphones' cameras were used to collect the data. With a 1/1.72 inch sensor size and Optical Image Stabilisation (OIS), the Vivo V21 camera produces high-quality images. Its 64MP sensor also enables Its outstanding image quality results from the sensor size and 0.8 µm pixel size as well as from modern image processing techniques. With pixel sizes of 0.64 µm and a sensor size of 1/2.76 inches, the Redmi Note 11 smartphone is equipped with a 50MP Samsung JN1 sensor. This high-resolution sensor combined with a wide aperture (f/1.8) guarantees outstanding light sensitivity and image clarity, which qualifies for obtaining comprehensive images needed for exact disease management insights in tea gardens.

### 4.2 Data preprocessing

Data preprocessing is an essential step in the development of machine learning models since it has a direct impact on the model's ability to acquire additional information from the input data. Data normalization, image resizing, and noise reduction were used in preprocessing of the dataset.

#### 4.2.1 Data labelling

In the first stage of data preparation, we carefully categorized the images and assigned each one to the suitable class or category. Supervised learning models require labeled data for training and optimization, as the labels enable the models to learn and make accurate predictions.

#### 4.2.2 Resizing

Our image has been reduced to 1024 × 1024 pixels from 2296 × 4080 pixels. To maintain computational efficiency, consistent dimensions of images and enabling the model to process data without additional complexity, we have resized the dataset. This ensures that every image is the same size and that the network may learn the same attributes from every one of them.

#### 4.2.3 Noise reduction

Gaussian smoothing is used for image denoising. This is done to eliminate any noise from the images that can impede CNN training process.

#### 4.2.4 Image segmentation

To improve the overall quality of the dataset, we adjusted the cropping as needed to remove undesired background items.

#### 4.2.5 Data augmentation

During our tea garden leaf image collection, we were unable to acquire an equal amount of data for each class, resulting in an imbalance. Deep learning is the foundation of our model, thus lots of images were required. A couple of images in a class could influence the accuracy of the categorization mechanism. Improving our experimental findings depends on a lot of data during the deep learning training period. We thus applied the image augmentation method to improve the count of training images. Zoom (probability=0.6, min_factor=1.1,max_factor=1.5),flip_left_right(probability=0.6),flip_top_bottom(probability=0.6), Rotate (probability=0.6, max_left_rotation=10, max_right_rotation=20). Among the augmented images are:

Selecting the optimal parameters for an augmentation technique is challenging [17]. We had to repeatedly perform the augmentation process to determine the optimal parameters that was challenging for us.

#### 4.2.6 Deep learning model validation

Our goal was to present a model using deep learning that would efficiently train the dataset to achieve cutting-edge results. A thorough assessment using a dataset is necessary for the deep learning algorithm to be validated. Deep learning models have each node as a processing unit and create related layers of nodes. The output layer produces the final product while the input layer receives data. Hidden layers with primary processing power for a neural network lie sandwiched between these inputs and outputs layers. In visual data analysis, deep learning models have developed dramatically in areas including image or video categorization, object detection, including natural language processing. The deep learning model uses an ordered five-stage procedure that includes data pre-processing, training of the models, data segmentation, evaluation of performance utilizing a validation set and finally, model testing on an entirely different test set. This methodical approach aids in verifying the model's ability to adapt to new data and its dependability in generating accurate results.

### 4.3 Model description

The proposed model, called TeaNet: Automated Classification of Tea Leaf disorders Using Convolutional Neural Networks, is made specifically to categorise images into four groups related to tea leaf disorders. The first layer of the architecture is an input layer that can hold RGB images with the form (224, 224, 3). Five convolutional blocks come second. Following a BatchNormalization layer and a Max Pooling2D layer using a pool size and stride of (2, 2), each of the first four blocks comprises of a Conv2D layer with ``same'' padding and ReLU activation". Whereas the third and fourth blocks contain 128 filters apiece, the first two blocks have 64 filters. The sixth block comprises BatchNormalisation and Max Pooling 2D layers after a Conv2D layer with 256 filters. The model consists of fully connected layers after the convolutional blocks, with the Flatten layer being the first to convert the 2D matrices into a 1D vector. A Dense layer with 512 units and ReLU activation comes next; a Dropout layer with a 0.5 dropout rate helps to minimise overfitting; yet another Dense layer with 256 units and ReLU activation follows. One additional output Dense layer (four units) using softmax activation for multiclass classification is added before the final Dropout layer (five dropout rate). The model is assembled with the ``accuracy'' metric, the ``adam'' optimizer, and a ``sparse_categorical_crossentropy'' loss function. Batch normalization and dropout layers improve training and lessen overfitting, whereas convolutional layers with fully connected layers efficiently combine for classification and feature extraction.

The model consists of 7113,284 trainable parameters, which significantly contribute to its capacity to learn complex features. Additionally, the model's size, calculated based on 32-bit floating-point precision, is approximately 27.14 MB. Large models may overfit on small datasets if not regularized properly, leading to inefficient use of resources. The number of parameters and model size must be carefully balanced to match the available computational resources and the specific application requirements [18].

### 4.4 Evaluation metrics

Deep learning and classification applications frequently use an assessment matrix including metrics include accuracy, recall, precision, and the F1-score. These indicators are crucial for evaluating how well classification models work. Each of them is broken down mathematically in [[19], [20], [21], [22]]:

(1) $$\text{Accuracy}=\;\frac{\text{TP}+\text{TN}}{\text{TP}+\text{TN}+\;\text{FP}+\text{FN}}$$

(2) $$\text{Precision}=\;\frac{\text{TP}}{\text{TP}+\;\text{FP}}$$

(3) $$\text{Recall}=\;\frac{\text{TP}}{\text{TP}+\;\text{FN}}$$

(4) $$F1\;\text{Score}=\;2\;\times \;\frac{\text{precision}\;\times \;\text{recall}}{\text{precision}+\;\text{recall}}$$

### 4.5 Confusion matrix

A confusion matrix is essential for assessing the effectiveness of a classification model, particularly when numerous classes are involved, as it provides insight into the degree to which the model's predictions match the actual class labels for various groupings [23]. Consequently, this matrix is crucial in identifying various groups since it defines their advantages and disadvantages concerning efficiency [[24], [25], [26]]. The confusion matrix allows data scientists to analyze student performance in a particular course, identify areas for development, and make well-informed decisions by classifying forecasts such true positives, true negatives, false positives and false negatives. Fig. 7 shows TeaNet model's confusion matrix for the dataset including tea plant illness categorization. This amazing accomplishment highlights how precisely TeaNet architecture can classify tea plant diseases. Table 8 shows that the model performed well in disease classification, with an accuracy rate of 98 %. This outstanding performance highlights the model's significant generalizing capacity, therefore demonstrating its relevance for pragmatic applications from fresh data. Using this dataset, in the upcoming years, we will thoroughly examine advanced deep learning models to determine the best strategy for practical application.

In the future, we seek to develop a consumer-focused smartphone application that uses artificial intelligence (AI) and machine learning techniques for image processing to help identify illnesses of tea plants like tea leaf blight, tea red scab, tea red leaf spot (Table 3, Table 3, Table 4, Table 5, Table 6, Table 7).

**Table 2:** Information of the dataset.

Name of class	No. of original data	No. of augmented data
Tea Leaf Blight	509	1000
Tea Red Leaf Spot	561	1000
Tea Red Scab	620	1000
Healthy	2270	1000
**Total**	**3960**	**4000**

**Table 3:** Attribute of original dataset.

Title	Description
Total Images	3960
Image Dimension	1024 × 1024
Color Gradings	RGB
Data Formats	JPG

**Table 4:** Augmentation techniques and their respective parameters.

Augmentation techniques	Parameters
Zoom	probability=0.6, min_factor=1.1,max_factor=1.5
flip_left_right	probability=0.6
flip_top_bottom	probability=0.6
Rotate	probability=0.6, max_left_rotation=10, max_right_rotation=20

**Table 5:** Split Dataset according to training and testing.

Dataset	Training	Testing
Tea Leaf Blight	700	300
Tea Red Leaf Spot	700	300
Tea Red Scab	700	300
Healthy	700	300
Total	2800	1200

**Table 6:** Model's layers and architecture.

Layer Type	Output Shape	Activation	Kernel Size / Pooling
Input layer	(224, 224, 3)	–	–
Conv2D	(222, 222, 32)	ReLU	(3,3)
MaxPooling 2D	(111, 111, 32)	–	(2,2)
Conv2D	(109, 109, 64)	ReLU	(3,3)
MaxPooling 2D	(54, 54, 64)	–	(2,2)
Conv2D	(52, 52, 64)	ReLU	(3,3)
MaxPooling 2D	(26, 26, 64)	–	(2,2)
Conv2D	(24, 24, 64)	ReLU	(3,3)
MaxPooling 2D	(12, 12, 64)	–	(2,2)
Conv2D	(10, 10, 64)	ReLU	(3,3)
MaxPooling 2D	(5, 5, 64)	–	(2,2)
Conv2D	(3, 3, 64)	ReLU	(3,3)
MaxPooling 2D	(1, 1, 64)	–	(2,2)
Flatten	(64)	–	–
Dense	(64)	ReLU	–
Dense	(4)	Softmax	–

### 4.6 Analyzing performance using the best model

**Table 7:** Classification report of Tea leaf disease classification.

Class name	Precision	Recall	F-1 score
Tea leaf blight	99	94	97
Tea red leaf spot	100	100	100
Tea red scab	95	100	97
Healthy	99	99	99

## 5 Limitations & Challenges

**Fig. 2:**
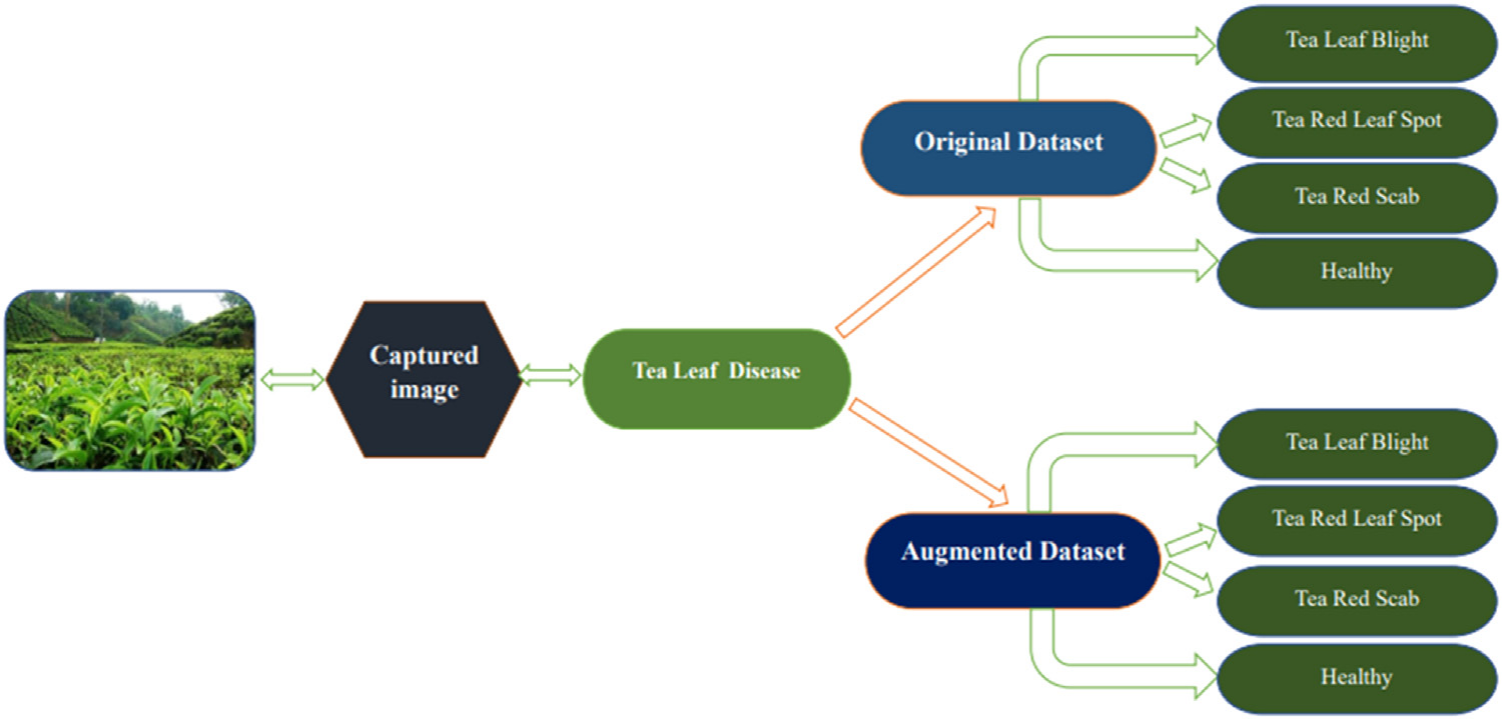
Organization of Tea Leaf disease dataset.

**Fig. 3:**
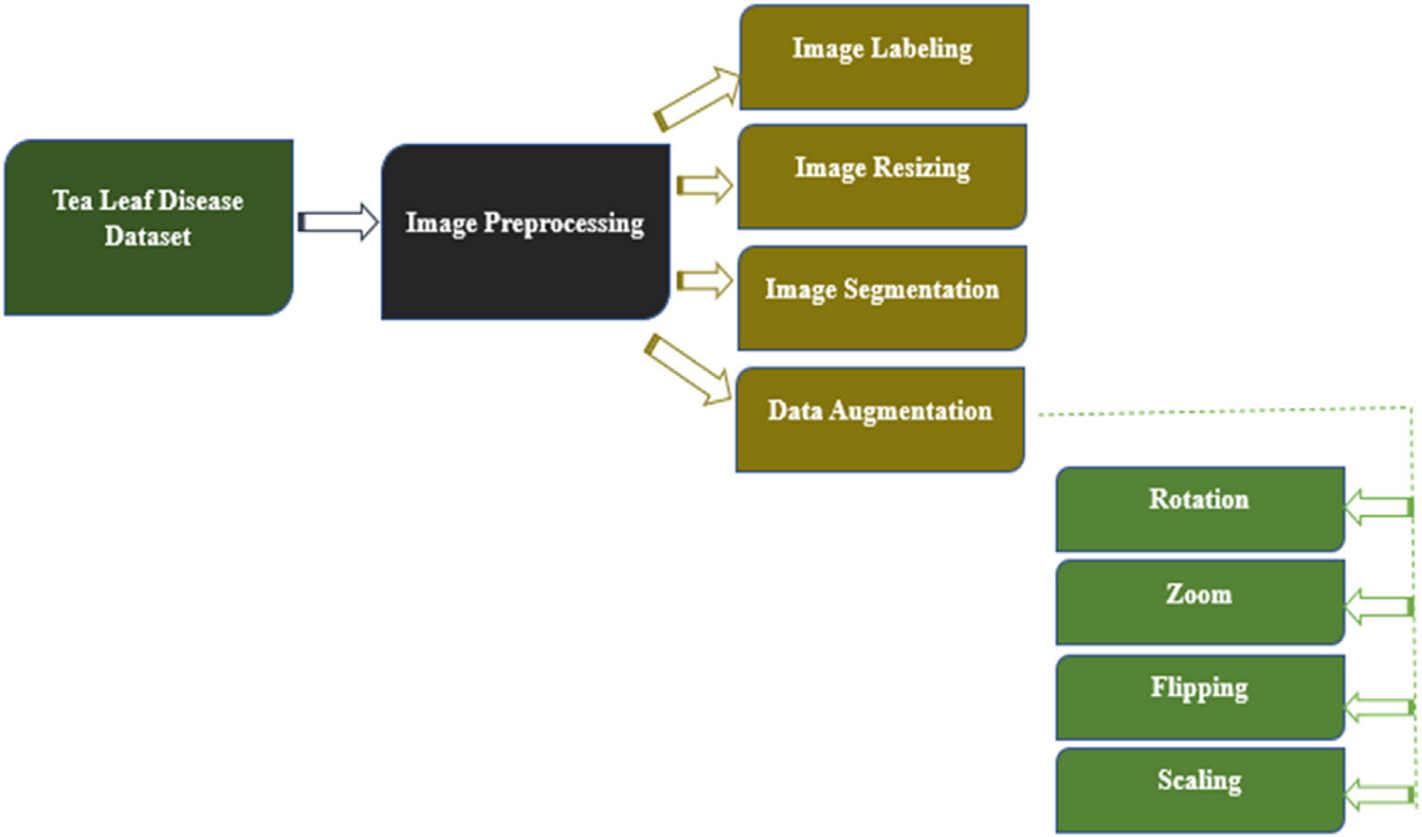
The steps in the proposed deep learning model's pre-processing.

**Fig. 4:**
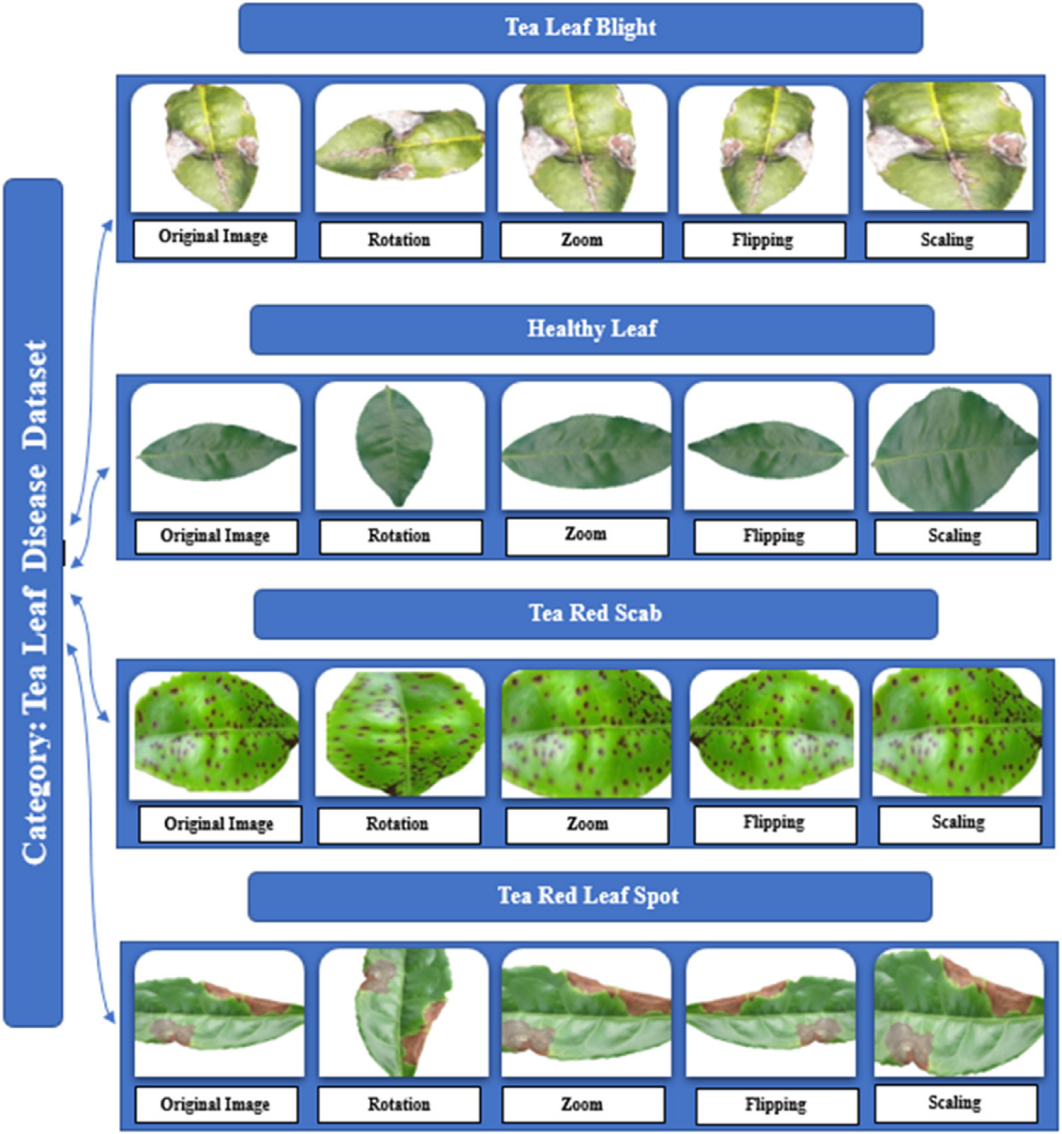
Data augmentation.

**Fig. 5:**
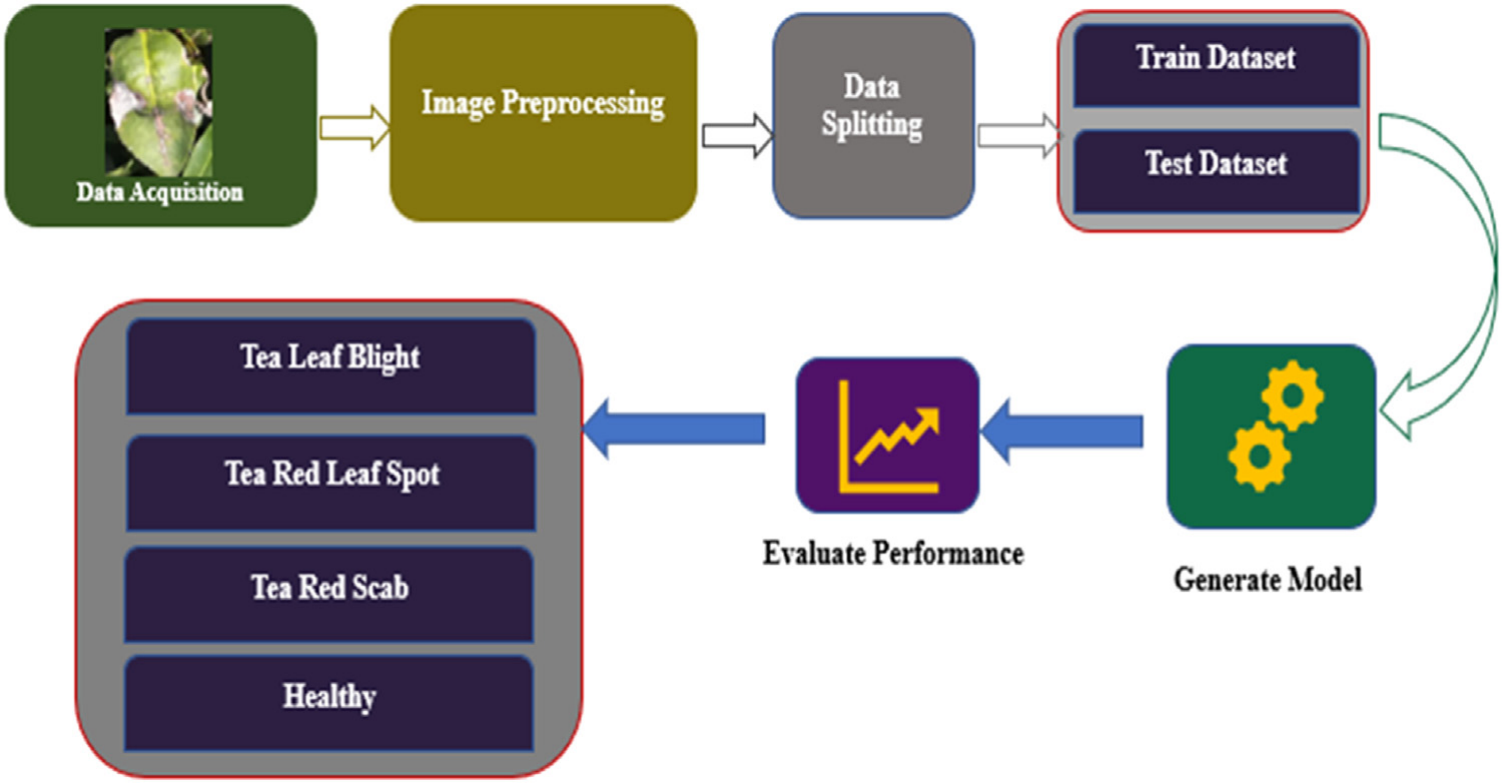
The working process for assessing tea leaf disease classification.

**Fig. 6:**
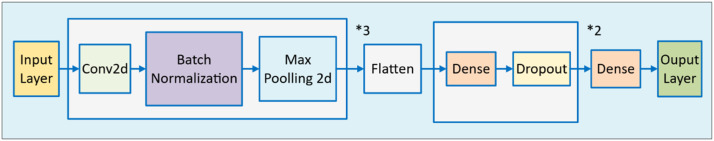
TeaNet architecture.

**Fig. 7:**
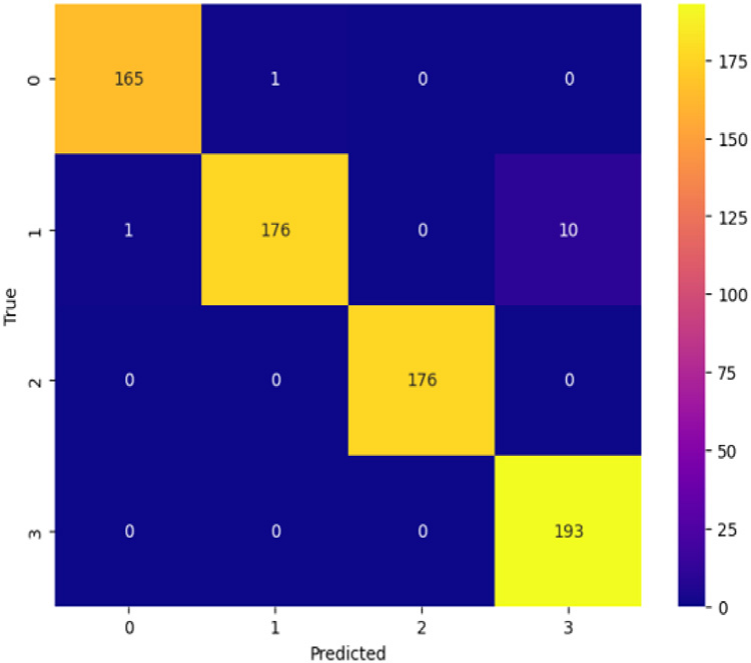
Tea-Net model's confusion matrix.

**Fig. 8:**
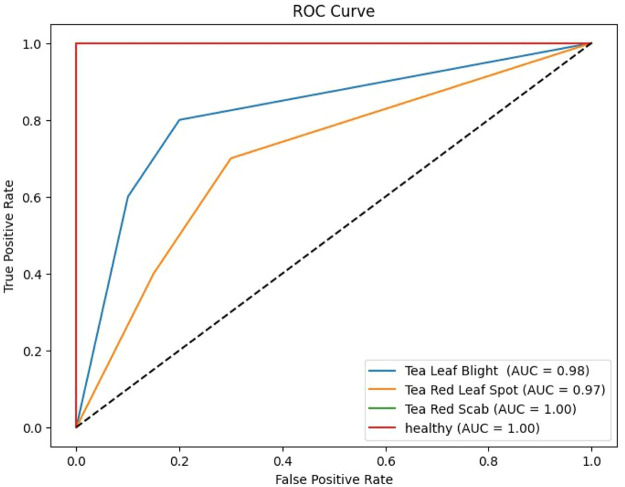
ROC-AUC curve.

This dataset primarily pertains to and is largely focused on tea leaf disease classification, making it unsuitable for classifying other plant diseases. This study faces several limitations and challenges including the reliance on smartphones for data collection, which may impact image quality and uniformity. The dataset is limited to only specific diseases, such as Tea Red Scab, Tea Red Leaf Spot, and Tea Leaf Blight, along with healthy tea leaves, narrowing the scope of the research. Additionally, the data was collected during two seasons and in specific locations, which may still introduce temporal and geographical biases, thereby limiting the broader applicability of the findings. Moreover, as there are no nearby tea gardens, we had to travel far from Dhaka to collect the dataset, which added significant logistical challenges (Fig. 1, Fig. 2, Fig. 3, Fig. 4, Fig. 5, Fig. 6, Fig. 7, Fig. 8).

## 6 Comparative Analysis with Existing Works

**Table 8:** Comparative analysis of our dataset in CNN model with existing dataset.

Research Paper	Dataset	Number of original images	Model	Accuracy
**This paper**	**Tea Leaf Disease Dataset**	**3960**	**Custom CNN**	**98 %**
[27]	Tea Leaf Disease Dataset	80	CNN	90.23 %
[28]	Tea Leaf Disease Dataset	3810	Custom CNN	90.23 %
[29]	Tea Leaf Disease Dataset	144	Improved CNN	92.25 %

## Ethics Statement

None of authors of this work have examined to any animal or human studies. Although the used datasets are publically available, following the proper referencing policies is still crucial.

## CRediT Author Statement

**Rimon:** Visualization, Conceptualization, Data curation, Methodology, Validation, Writing. **Sajib Bormon:** Visualization, Methodology, Data curation. **MD Hasan Ahmad:** Methodology, Validation, Writing. **Sohanur Rahman Sohag:** Writing, Methodology, Data curation, review, editing. **Amatul Bushra Akhi:** Supervision, Writing, review. **Mohammad Shorif Uddin:** Writing, review and editing*.*

## Future Work

In the future, we aim to enhance this work by implementing practical applications through real-world testing and deployment in various environmental conditions. We also plan to expand the dataset and model capabilities to detect a broader range of tea leaf diseases and apply the approach across other tea-growing regions. These advancements will improve the model's generalizability and practical relevance, supporting broader applications in tea plant health monitoring and disease management.

## Data Availability

Mendeley DataAdvanced Tea Crop Disease Study: High-Resolution Dataset for Precision Agriculture and Pathological Insight (Original data).
